# Fluorescence in Situ Hybridization (FISH) for Detecting Anaplastic Lymphoma Kinase (*ALK*) Rearrangement in Lung Cancer: Clinically Relevant Technical Aspects

**DOI:** 10.3390/ijms20163939

**Published:** 2019-08-13

**Authors:** Zhenya Tang, Lu Wang, Guilin Tang, L. Jeffrey Medeiros

**Affiliations:** 1 Department of Hematopathogy/Clinical Cytogenetics Laboratory, the University of Texas MD Anderson Cancer Center, Houston, TX 77030, USA; 2 Department of Pathology, St. Jude Children’s Research Hospital, Memphis, TN 38105, USA

**Keywords:** ALK, fluorescence in situ hybridization (FISH), immunohistochemistry (IHC), next generation sequencing (NGS), cutoff value, discordant result, incidental finding

## Abstract

In 2011, the Vysis Break Apart ALK fluorescence in situ hybridization (FISH) assay was approved by the United States Food and Drug Administration as a companion diagnostic for detecting *ALK* rearrangement in lung cancer patients who may benefit from treatment of tyrosine kinase inhibitor therapy. This assay is the current “gold standard”. According to updated ALK testing guidelines from the College of American Pathologists, the International Association for the Study of Lung Cancer and the Association for Molecular Pathology published in 2018, ALK immunohistochemistry is formally an alternative to ALK FISH, and simultaneous detection of multiple hot spots, including, at least, *ALK*, *ROS1*, *RET*, *MET*, *ERBB2*, *BRAF* and *KRAS* genes is also recommended while performing next generation sequencing (NGS)-based testing. Therefore, ALK status in a specimen can be tested by different methods and platforms, even in the same institution or laboratory. In this review, we discuss several clinically relevant technical aspects of ALK FISH, including pros and cons of the unique two-step (50- to 100-cell) analysis approach employed in the Vysis Break Apart ALK FISH assay, including: the preset cutoff value of ≥15% for a positive result; technical aspects and biology of discordant results obtained by different methods; and incidental findings, such as *ALK* copy number gain or amplification and co-existent driver mutations. These issues have practical implications for ALK testing in the clinical laboratory following the updated guidelines.

## 1. Introduction

Lung cancer is the most common cause of cancer-related deaths in the United States [1] and in many other countries [2,3]. An estimated 142,670 deaths from lung cancer will occur in 2019 in the United Sates [1] and over 1.7 million patients died of lung cancer worldwide in 2018 [2,3]. The development of molecularly targeted therapeutics, such as tyrosine kinase inhibitors (TKI) directed against epidermal growth factor receptor (*EGFR*) mutations (e.g., erlotinib, gefibinib and osimertinib) and anaplastic large-cell lymphoma kinase (*ALK*) rearrangement (e.g., crizotinib, certinib, alectinib and brigatinib), has improved outcomes of lung cancer patients with these mutation [4,5,6,7]. However, only 3−7% of non-small cell lung cancers (NSCLC) carry *ALK* rearrangement, which is mostly driven by an in-frame fusion of echinoderm microtubule associated protein like 4 (*EML4*) and *ALK*, both located on 2p23 and approximately 12 Mb apart. Patients with lung cancer that carry EML4-ALK fusion respond to tyrosine kinase inhibitors (TKI), such as crizotinib, and therefore unambiguous detection of *ALK* status in a lung cancer specimen is critical for a treatment decision and predicts the outcome of the affected patient.

Crizotinib was the first TKI approved by the United States Food and Drug Administration (FDA) as a targeted therapy for patients with *ALK* rearranged NSCLC in 2011. Simultaneously, the FDA approved the Vysis ALK Break Apart fluorescence in situ hybridization (FISH) Probe Kit (Abbott Molecular) as a companion diagnostic test to identify the patients most likely to benefit from crizotinib therapy. This commercial kit has been considered to be a “gold standard” for clinical testing for *ALK* rearrangements, but there are challenges raised about this assay regarding its analytical sensitivity and specificity, accuracy of test results, and high expense as a screening test, as well as the development of other methods/technologies, such as immunohistochemistry (IHC) and next generation sequencing (NGS) that are currently available to assess *ALK* status.

In this review, we attempt to concisely discuss several clinically relevant technical issues that are sometimes troublesome for clinical laboratories performing this test, including: the uncommon two-step (50- to 100-cell) analysis approach; the 15% cutoff value for a positivity; discordant results obtained by different methods/technologies; and incidental findings, such as *ALK* copy number gain and amplification or coexistent driver mutations. We also propose an algorithm for *ALK* testing in a clinical laboratory.

### 1.1. The Uncommon Two-Step (50- To 100-Cell) Analysis Approach

**Figure 1:**
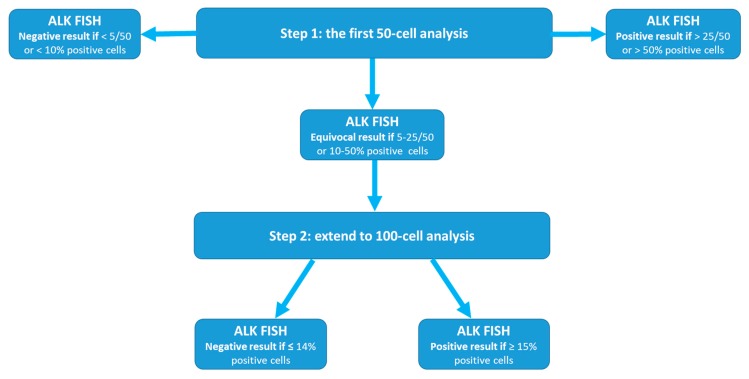
Schematically illustration of the two-step (50- to 100-cell) analysis approach of the Vysis ALK (anaplastic lymphoma kinase) Break Apart FISH (fluorescence in situ hybridization).

The FDA-approved Vysis ALK Break Apart FISH probe consists of a 3′*ALK* probe of approximately 300 kb labeled in SpectrumOrange and a 5′*ALK* probe of about 442 kb labeled in SpectrumGreen. The 3′*ALK* and 5′*ALK* probes are located centromerically and telomerically to the *ALK* breakpoint, respectively [8]. According to the Vysis ALK Break Apart FISH evaluation guide for NSCLC tissue specimens and the summary of safety and effectiveness data provided by Abbott Molecular [8,9], the protocol is a two-step (50- to 100-cell) analysis approach (Figure 1), as follows:

Step 1: After hybridization and location of target area for analysis, 50 nuclei are counted initially and test results are categorized as follows: 1). Negative if <5 cells (<5/50 or <10%) have positive signals; 2). Positive if >25 cells (>25/50 or >50%) are positive; 3). Equivocal if 5 to 25 cells (or 10% to 50%) are tested with positive signal(s).

Step 2: for all samples with an equivocal result, a second reader needs to evaluate an additional 50 nuclei and the results for all 100 nuclei by the first and second readers are added together and a percentage is calculated [10]. 1). Negative if <15% (<15/100) nuclei have positive signals; and 2). Positive if ≥15% (≥15/100) nuclei have positive signals.

This uncommon two-step approach is different from the current American College of Medical Genetics and Genomics (ACMGG) technical standards and guidelines for FISH analysis [11] where usually a one-step analysis of 200-cells for both liquid (bone marrow, peripheral blood, etc.) and tissue specimens is recommended. Although the ACMGG has stated the analytical criteria for FDA-approved probes supersede the general rules listed in its standard and guidelines [11], we believe that this uncommon ALK FISH test approach has advantages. The first of these is the unavailability of standards and guidelines from a professional and/or scientific society for tissue FISH testing, or more specifically for ALK FISH testing in NSCLC, and the approval of this assay by the FDA. In 2011, the ACMGG FISH guidelines [12] were applied only for constitutional disorders and the Association for Molecular Pathology (AMP)/ACMGG guidance only for hematologic disorders (only liquid specimens) [13]. Secondly, previous studies, especially the first pivotal clinical trials leading to the FDA approval of crizotinib [14,15], were successful in identifying patients for targeted therapy by adopting this approach. Third, a 50-cell or 100-cell analysis is obviously more cost-effective than a 200-cell analysis, providing the lower counts come to the same conclusion as a 200-cell count for the same specimen. Lastly, theoretically the analysis of more cells may “dilute” the percentage of positivity if the number of positive cells is actually limited in a specimen.

Camidge et al. [16] has shown that analysis of ≥60 cells from ≥4 selected tumor areas of a NSCLC specimen can reach 100% specificity and 100% of sensitivity for *ALK* rearrangement testing, although the Vysis ALK Break Apart FISH probe set used in their study is slightly different for coverages of both 5′*ALK* and 3′*ALK* probe from the one approved by the FDA. These authors also emphasized that analysis of ≥60 cells from ≥4 selected tumor areas is considered as a minimum requirement for accurately testing the status of *ALK* in NSCLC specimens. Therefore, this two-step approach seems to be rational and practical for clinical laboratory practice [17,18]: a 50-cell analysis (step 1) to identify specimens with high percentages of *ALK* rearrangements (>25/50 or >50%) and then another 50-cell analysis (step 2) for specimens with equivocal results. However, there are apparent drawbacks of this uncommon two-step approach. First, specimens with a definite positive result at the first step (>25/50 or >50%) and specimen tested as positive during the second step have been analyzed differently for the total cell number and tumor areas. The accuracy of percentages of positive cells in these specimens can fluctuate when they are analyzed the same way (e.g., 200-cell read). Therefore, categorizing NSCLC patients into “high *ALK* rearranged” and “low *ALK* rearranged” based on the percentages by this approach and correlating with therapy response and clinical outcomes should be interpreted cautiously [19,20]. Secondly, specimens with an equivocal result with 15 (≥15/50) to 25 (≤25/50) positive cells in the first step will be definitely considered as positive (≥15/100 or ≥15%), no matter whether any additional positive cells will be identified in the second step. The rationale for the more extensive analysis in these specimens is not explained or understood [8,9]. A more cost-effective way could be to count to 100 cells only for those specimen with 5 to 14 positive cells in the first step.

### 1.2. The Cutoff Value of ≥15/100 or ≥15% for a Positive Test Result

Although the ≥15% result has been widely applied for clinical decisions, including patient selection for targeted therapy and the prediction of treatment response, this cutoff value for a positive *ALK* rearrangement in a NSCLC specimen was initially not defined by a clinical end point. Instead, it was established based on an analytical assessment of the background signals (“background noise”) [16,21,22]. Shaw et al. [21] and Rodig et al. [22] first applied this value as a cutoff for a positive *ALK* rearrangement in their studies, but it is not disclosed how this cutoff value was established. Camidge et al. [16] reported the validation of this cutoff value in detail. In their report, 17 previously tested ALK-positive (ALK+) and 15 ALK-negative (ALK−) NSCLC specimens were thoroughly analyzed to document ALK FISH signal pattern(s) in all types of cells of each specimen, including ALK+ tumor cells, ALK− tumor cells and their adjacent normal areas. An average of 53.8% (range 22.3−86.6%) of ALK+ tumor cells, 6% (range 3.5−9.5%) of ALK− tumor cells, 6.8% (range 2.1−11.1%) of adjacent non-tumor cells in ALK+ specimens, and 5.3% (range 0.7−11.2%) of adjacent non-tumor cells in ALK− specimens exhibited positive ALK FISH signal patterns. Therefore, a non-overlapping area of 12% to 21% positivity exists between the ALK+ tumor cells and background noise (up to 11% of positivity obtained from both the ALK− tumor cells and non-tumor cells). The cutoff value of ≥15% falls into this non-overlapping area and is thought to accurately distinguish true positive results from background noise caused by assay variability. This cutoff value was first adopted and correlated with response in the first crizotinib clinical trial [14,15].

Although it is currently widely applied in almost all clinical laboratories performing the Vysis ALK Break Apart FISH assay in the United States, the rationale of using this cutoff value has been challenged for at least three reasons:

1). This cutoff value is based on analysis of a limited number of NSCLC cases from a single institution [16]. All 13 *ALK* rearrangement positive cases included in the study showed definite positivity with high percentages of positive cells (e.g., ≥28% (13/13), >40% (11/13) and >50% (9/13), respectively). No cases with a low number of positive cells (e.g., of 15–20% or even lower) was included. Interestingly, an Italian group [23] has validated the Vysis ALK Break Apart FISH probe and Dako ALK FISH Probe (now a subsidiary of Agilent, Santa Clara, CA) in a way similar to that recommended by the ACMGG [11] in 2015. The cutoff value that they have obtained from ALK negative panel of controls is 14.9% (mean ± 3 SD), almost as the same as that suggested by Abbott Molecular.

2). In all 13 positive cases, the *ALK* rearrangement positive cells were distributed diffusely in all tumor areas tested, and the authors concluded that *ALK* rearrangement is a diffuse event and the exact percentages of positive cells have little impact on outcome of ALK inhibitor treatment. The authors in another study further emphasized this observation [17]. However, more and more studies from other research groups have shown that a good response to crizotinib therapy was observed in some patients with NSCLC in which the percentage of *ALK* rearrangement positive cells was <15%, indicating the existence of low levels of *ALK* rearrangement and a focal, non-random distribution of positive cells in some NSCLC cases [24,25,26,27,28]. Soria et al. [19] performed a pooled meta- analysis of three large prospective crizotinib clinical trials for *ALK* positive NSCLC, designated as PROFILE 1005 [29], PROFILE 1007 [30] and PROFILE 1014 [31,32], to evaluate the relationship between the percentage of *ALK* rearrangement positive cells and clinical outcomes or crizotinib responses. Among 1958 ALK positive patients enrolled in these trials, the median percentage of *ALK* rearrangement positive cells was 58% (range 15−100%). The objective crizotinib response rates were 56% (*n* = 700/1246), 55% (*n* = 725/1312) and 38% (*n* = 25/66) for patients with *ALK* rearrangement positive cells at a percentage of >20%, ≥15% and 15−19%, respectively, implying that this 15% cut-off is clinically valid for diagnosis of *ALK* rearrangement and patient selection for targeted therapy. At the same time, this meta-analysis also indicated that NSCLC patients with a higher percentage of *ALK* rearrangement positive cells tend to respond better to the crizotinib therapy [19,32].

3). Due to the two-step (50- to 100-cell) analysis approach, a range of equivocal results, also called “a safe zone for reporting”, is eliminated. All cases with a percentage of *ALK* rearrangement positive cells in the non-overlapping area of 12% to 21% can be extremely challenging to a laboratory director who is interpreting the results. Therefore, terms such as “borderline negative result (i.e., 10–14%)” and “borderline positive result (i.e., 15–24%)” have been suggested for further analysis of these cases by different methods/technologies (e.g., IHC, NGS or other methods) by others [19,33,34,35].

### 1.3. Discrepancies among Results Obtained by Other Methods

Prior to its approval as a companion diagnostic kit for testing *ALK* rearrangement in NSCLC, the ALK FISH test (Vysis ALK Break Apart FISH Probe Kit) was already being challenged by other methods/technologies, especially ALK immunohistochemistry (IHC) [21,22,36]. Others have reported that IHC is equally efficient, but more cost-effective than ALK FISH for screening ALK status in NSCLC specimens. However, discrepancies in results obtained by FISH and IHC, such as ALK FISH+/ALK IHC− or ALK FISH−/ALK IHC+, have been shown in some cases [24,37,38,39,40]. In the past decade, some new and better anti-ALK antibodies, as well as automation of IHC procedures, have been developed, and the new generation ALK IHC is considered as sensitive as ALK FISH for detecting *ALK* rearrangement [41,42,43,44]. In 2015, the FDA approved the VENTANA ALK (D5F3) CDx Assay (Roche Diagnostics) for a qualitative detection of ALK protein in NSCLC tissue specimens to identify patients eligible for treatment with crizotinib [45,46].

Many groups have applied RT-PCR to detect *ELM4-ALK* fusion, the most common form of *ALK* rearrangement at RNA level [47,48,49]. According to their studies, the *ELM4-ALK* RT-PCR is both highly sensitive and specific for detection of *ALK* rearrangement in NSCLC cases. The concordances between this method and ALK IHC or ALK FISH is approximately 95–100% [47,48,49,50]. Next-generation sequencing (NGS) based methods are also becoming widely utilized in the detection of *ALK* rearrangement. NGS-based methods have identified many *ALK* rearrangement positive cases with an atypical ALK FISH signal pattern and even in cases negative by ALK FISH [51,52,53,54]. However, NGS-based methods differ in targets (DNA or RNA), enrichment of target (amplicon-based or capture-based), tumor burden, analytical sensitivity and specificity [55,56]. Other methods/techniques, such as multiplexed PCR-based assay [57] and droplet digital PCR [58,59], as well as NanoString technology [60,61], also have been applied to detect *ALK* rearrangement with success.

The discrepancies among results for *ALK* rearrangement obtained by different methods have been reported in many studies, but the frequency of discrepancy has been variable. For example, von Laffert et al. [62] reported a complete concordance between ALK FISH, ALK IHC and RT-PCR in 15 NSCLC cases. In contrast, Matterson et al. [63] reported 64% agreement between ALK FISH and ALK IHC (Ventana) (FISH+/IHC+ in nine cases; FISH+/IHC− in four cases; FISH-/IHC+ in five cases); 54% agreement between ALK FISH and ALK IHC (Dako) (FISH+/IHC+ in eight cases; FISH+/IHC− in five cases; FISH-/IHC+ in three cases); and 67% agreement between ALK IHC (Ventana) and ALK IHC (Dako) (Ventana+/Dako+ in 10 cases; Ventana+/Dako- in four cases; Ventana−/Dako+ in one case), respectively. Interestingly, among six (3%) cases with high ALK expression (mRNA level) tested by Affymetrix Gene Microarray, only three were positive by ALK FISH (two cases) or ALK IHC (Ventana). Gao and colleagues [64] also reported that the signal patterns in ALK FISH positive cases may be associated with the discordance with ALK IHC. In their study, all 27 ALK FISH positive samples with a split signal pattern (1R1G1F) were ALK IHC positive (100% concordance), whereas three of 21 ALK FISH positive samples with a 5′ALK deletion signal pattern (1R1F) were ALK IHC negative (11% discordance); two of these cases were negative *ALK* rearrangement as tested by a targeted NGS assay. Dacic et al. [64] observed an even larger discordance between ALK FISH and NGS testing, e.g., 20% (3/15) for ALK FISH positive cases with a signal pattern of 1R1G1F; 60% (6/10) for ALK FISH positive cases with a signal pattern of 1R1F; and 100% (3/3) for ALK FISH positive cases with mixed signal patterns of 1R1G1F and 1R1F. The authors also stated that the signal patterns of ALK FISH showed no statistically significant association with crizotinib response, whereas NGS and ALK IHC positive cases were associated with a significantly higher response rate than NGS and ALK IHC negative cases (*p* = 0.016).

These discrepancies can be caused by multiple factors, such as technical and biological issues. Examples of technical issues include the analytical sensitivity and specificity of each method and pre-analytical factors such as the quality and preparation of tested specimens. Biologic issues include disproportion between *ALK* rearrangement and *ALK* transcription and/or ALK translation [65]; complex chromosomal aberrations involving *ALK* rearrangement that are beyond FISH detection; different hetero- or homo-dimerization properties and stabilities of various chimeric ALK proteins; and *ALK* overexpression through other mechanisms, such as gene amplification or mutations [33,34]. It is beyond the scope of this review to discuss all these methods and their advantages and disadvantages in detail. A brief comparison of various methods used for ALK status evaluation is summarized in Table 1. In general, NSCLC specimens with either a “borderline negative” or a “borderline positive” ALK FISH result are more prone to have a discordant result obtained by other methods [23,34,35,52,56,62,66,67]. Specimens with borderline ALK FISH results need to be tested using another method to verify the suspected ALK alteration so that unnecessary targeted therapy can be avoided in patients with a false positive test result, and patients with a false negative result still have the opportunity to be properly treated.

### 1.4. Development of a Diagnostic Algorithm for Detection of ALK Status

According to the updated molecular testing guidelines for the selection of lung cancer patients for treatment with targeted tyrosine kinase inhibitors recommended by the College of American Pathologists, the International Association for the Study of Lung Cancer and the Association for Molecular Pathology in 2017 [68], as well as the FDA having approved the VENTANA ALK (D5F3) CDx Assay (Roche Diagnostics) for a qualitative detection of ALK protein in NSCLC tissue specimens, both ALK FISH and the ALK IHC are considered as the first line methods for *ALK* rearrangement testing. The guidelines also state that there is no apparent superiority of ALK FISH over the ALK IHC or vice versa, implying that either one these tests can be performed currently. The guidelines also state that a borderline result by the ALK FISH and weak staining by the ALK IHC can be extremely challenging to interpret. In this circumstance, the guidelines emphasize that specimens with discrepancies, either ALK FISH+/ALK IHC− or ALK FISH−/ALK IHC+, should be further tested by another validated method, such as RT-PCR and NGS [68]. Although there is no NGS-based assay approved by the FDA as a first line method for determining ALK status yet, this powerful methodology, also called massively parallel sequencing, has been changing the diagnostic field dramatically, in both lung cancer and all other diseases. Generally, the capture-based DNA or RNA NGS assay is preferred to the amplicon-based NGS assays of DNA for their coverages of variant ALK fusions [68]. It has been speculated that NGS assays will become first line of methods for ALK status testing, together with testing many other mutations in lung cancer. The RT-PCR assay is mainly a standard practice for determining ALK status in many other countries [47,69,70].

**Figure 2:**
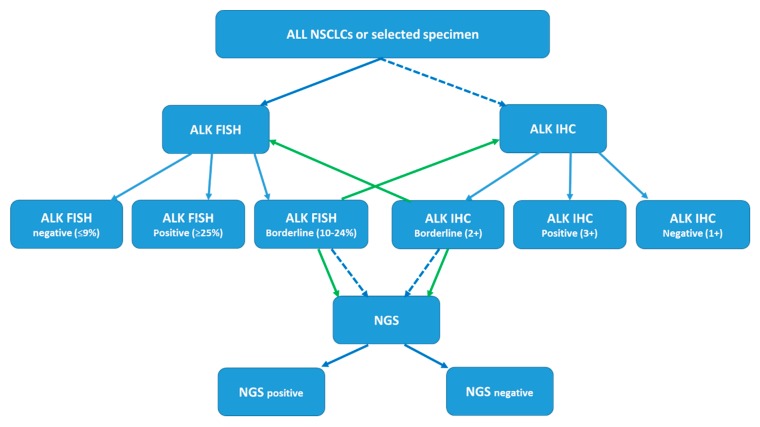
Proposed diagnostic algorithm for ALK status testing in lung cancer. For example, a specimen can be tested with ALK FISH (or ALK IHC) first. If a borderline result (either borderline negative or borderline positive) is obtained, the sample will be further tested with ALK IHC (or ALK FISH). If the ALK status is still uncertain, then targeted RNA-seq is applied and the final ALK status is determined based on the NGS result. For cases with discrepant results obtained by two methods, a third methodology, if available, should be applied to clarify the discrepancy. Solid blue arrow: proposed primary test methodology or predicted test results. Solid green arrow: proposed reflex test(s). Dashed blue arrow: alternative test methodology.

Therefore, laboratories providing ALK testing using one or more of the methods mentioned above need to develop a diagnostic algorithm based on the availability, accessibility, cost-effectiveness and on-site validation. For example, either the FDA-approved ALK FISH or ALK IHC can be the main methodology. If a “borderline” result is obtained in a specimen, a reflex test should be performed. For cases with discrepant results obtained by two methods, a third methodology, if available, should be applied, so that a clear result can be obtained for each specimen and the possibility of false positive or false negative results can be maximally reduced or even eliminated [69,71,72]. As an example, a proposed testing algorithm using the ALK FISH as the first line method and ALK IHC and NGS as the confirmatory test is illustrated (Figure 2). This can be modified according to the landscape and practical implementation issues in each laboratory.

### 1.5. Incidental Findings during ALK FISH Testing

#### 1.5.1. ALK Gene Copy Number Gain/Amplification

**Figure 3:**
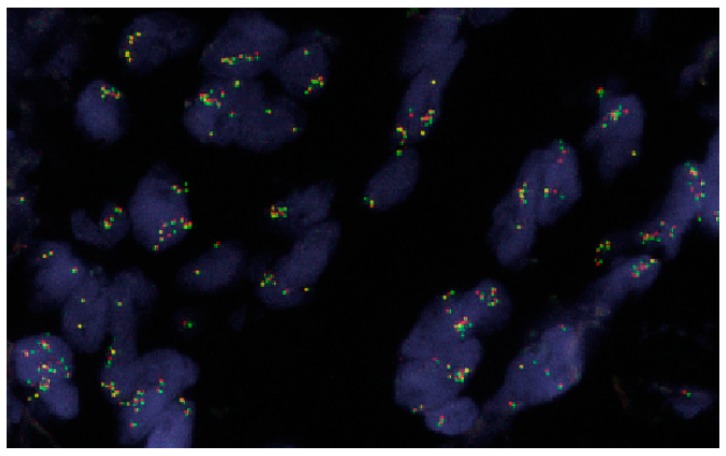
An example of *ALK* amplification in a lung adenocarcinoma case. The majority of tumor cells showed 6 to 20 copies of *ALK* gene or cluster *ALK* signals (60x).

*ALK* gene copy number gains (3−5 copies/cell), especially *ALK* gene amplification (>6 copies/cell and/or clusters of signals) (Figure 3) are well studied in other types of cancer, i.e., neuroblastoma, and are generally considered to be associated with aggressive tumor behavior and poorer outcomes [73]. In fact, *ALK* amplification is frequently detected in NSCLC and is more frequent than *ALK* rearrangement. In a study of 107 NSCLC cases reported by Salido et al. [74], only three (3%) cases exhibited *ALK* rearrangement, but 11 (10%) and 68 (63%) cases exhibited *ALK* amplification and copy number gains, respectively. In addition to the ALK break-apart probe, the authors also included CEP2 (a centromeric alpha-satellite probe specific for chromosome 2) in the FISH test to distinguish *ALK* gains/amplification from polysomy of chromosome 2. Interestingly, only two cases with *ALK* rearrangement were ALK–IHC+, whereas all other cases, including one case with *ALK* rearrangement and all 11 cases with *ALK* amplification were ALK IHC−. Preusser et al. [75] observed a similar frequency of *ALK* amplification, but also noticed that two cases with brain metastasis exhibited co-existent *ALK* rearrangement and *ALK* amplification in their study. The FDA-approved Vysis Break Apart ALK FISH probe does not include a control probe such as the CEP2 probe, but it can reflect the copy number of *ALK* in each specimen (Figure 3). Camidge et al. [17,18] reported that 19% of *ALK* rearrangement positive and 62% of *ALK* rearrangement negative NSCLC cases exhibited an increased *ALK* copy number (>3 copies per cell in >40% of cells). Peretti et al. [76] reported that *ALK* copy number gain/amplification is very common in smoker/former smoker compared to non-smoker NSCLC patients (74.2% versus 20.4%), which might provide a certain indication of genomic instability. Although the relationship between *ALK* copy number gain/amplification and sensitivity to crizotinib treatment is not established, as recommended by most studies, cases with *ALK* amplification should be further investigated [17,18,73,74,75,76]. It is important to emphasize that the definition of *ALK* copy number gain/amplification varies among these studies, and validated cutoff values need to be established in each individual laboratory.

#### 1.5.2. Co-Existent Driver Mutations

Others have reported in earlier studies that *ALK* rearrangements are mutually exclusive with other driver mutations, e.g., mutations in *EGFR* or *KRAS*, *ROS1* rearrangement, *RET* rearrangement or *MET* amplification [14,15,77]. Patients with one of these mutations might be excluded for *ALK* rearrangement testing at that time [16,17,68,78]. Recently, cases with coexistence of two or more driver mutations including *ALK* rearrangement have been reported [79,80,81], in part attributable to detection of multiple biomarkers from the same specimen and/or utilization of technologies, such as NGS, which detects multiple hotspots simultaneously. Therefore, a comprehensive genomic testing of many known driver mutations in NSCLC specimens, including at least *ALK*, *ROS1*, *RET*, *MET*, *ERBB2*, *BRAF* and *KRAS* genes, has been proposed if NGS based methodology is performed [68]. It seems likely that more cases with co-existing driver mutations will be identified. However, it remains unknown whether the co-existing driver mutations are from the same tumor cells, for which one targeted agent may be applied, or from different tumor cells representing a type of intratumoral heterogeneity, for which various specific agents for different targets may be necessarily applied simultaneously or one after another.

In summary, the FDA-approved Vysis Break Apart ALK FISH probe has served as the “gold standard” for assessment of ALK status in NSCLC specimens during the past decade and is still widely used in clinical laboratories in the United States. In this concise review, we have discussed practical issues related to several clinically relevant technical aspects, including: the pros and cons of the unique two-step (50- to 100-cell) analysis approach; the preset cutoff value of ≥15% for a positive result; technical causes and biology of discordant results obtained by different methods; and incidental findings during ALK FISH testing [68].

## 2. Conclusions

Both “borderline negative” and “borderline positive” ALK FISH results can be a huge challenge to correctly interpret. For these clinical scenarios, a reflex test should be available. Cases with discordant results obtained by different methods, e.g., ALK FISH and ALK IHC, should be further investigated, e.g. by NGS-based method(s). A test algorithm is strongly recommended for clinical laboratories where several tests for ALK status in NSCLC specimens are offered and/or available.

## Figures and Tables

**Table 1:** A brief comparison of methodologies being applied for evaluation of ALK status.

Methodologies	Biology	Partner Gene Dependent?	Potential False Negative Results by Biology
**ALK FISH**	*ALK* gene rearrangement; not for *ALK* mutation(s)	No	*ALK* rearrangement driven by cryptic and/or complex chromosomal abnormalities beyond FISH detection; abnormal ALK status by mechanism(s) other than *ALK* rearrangement
**ALK IHC**	Abnormal ALK protein expression caused by *ALK* rearrangement and/or mutation(s)	Yes (certain *ALK* rearrangement may have higher ALK protein expression than the others)	*ALK* rearrangement without high level ALK protein expression
**RT-PCR**	*ALK* fusion transcripts (RNA level); not for *ALK* mutation(s)	Yes	unknown partner gene(s)/fusion points for *ALK* rearrangement
**NGS-based targeted DNA-seq**	*ALK* mutation(s) +/− rearrangement; depending on the platform used	Not necessary	variant fusion points outside the targeted capture region
**NGS-based targeted RNA-seq**	*ALK* rearrangement +/− mutation(s)	No	extremely rare

Abbreviations: ALK gene: anaplastic large-cell lymphoma kinase gene; FISH: fluorescence in situ hybridization; IHC: immunohistochemistry; NGS: next generation sequencing; RT-PCR: reverse transcription-polymerase chain reaction; seq: sequencing.
